# Real-world analysis of community colorectal neoplasia screening based on stool DNA methylation detection

**DOI:** 10.3389/fonc.2025.1562698

**Published:** 2025-06-06

**Authors:** Ming-sheng Fu, Shu-xian Pan, Xun-quan Cai, Ya-wen Cao, Li-yuan Jin, Qin-cong Pan

**Affiliations:** ^1^ Department of Gastroenterology, Shanghai Fifth People’s Hospital Fudan University, Shanghai, China; ^2^ Department of Nephrology of Shanghai Fifth People’s Hospital Fudan University, Shanghai, China; ^3^ Department of Public Health, Shanghai Minhang District Maqiao Community Health Service Center, Shanghai, China

**Keywords:** colorectal neoplasia, stool DNA methylation detection, fecal occult blood testing, screening, colorectal cancer

## Abstract

This study aimed to assess the effectiveness of stool DNA methylation detection (sDNAMD) in improving colorectal neoplasia (CN) detection rates and colonoscopy compliance in a real-world community setting. Between July 1, 2023, and June 30, 2024, residents aged 50–75 from Maqiao Town, Minhang District, Shanghai, were invited to participate in a CN screening program. Participants were randomly assigned to one of three groups: high-risk questionnaire + fecal occult blood test (HRFO), high-risk questionnaire + FOBT + sDNAMD (HRFOsD), or sDNAMD only (sDNA). Colonoscopy was performed based on initial screening results, and the number of individuals undergoing colonoscopy, along with results, were recorded to calculate compliance and CN detection rates. The HRFOsD group exhibited a significantly higher colonoscopy compliance rate (93.7%) compared to the HRFO (32.6%) and sDNA (73.5%) groups (P<0.0001). Residents with negative FOBT, negative sDNA results, or those who did not undergo sDNA testing did not undergo colonoscopy. In the HRFOsD group, the CN detection rate was higher in females compared to males. Compared to the HR and FOBT+ group, the CN detection rate was significantly higher in the LR and FOBT+ and sDNA+ group. Adding sDNAMD to the high-risk questionnaire and FOBT screening led to a notable increase in both colonoscopy compliance and CN detection rates. The conclusion is that adding stool DNA methylation detection to the community FOBT screening program can significantly improve colonoscopy compliance and colorectal neoplasia detection rates among community residents.

## 1 Introduction

Colorectal cancer (CRC) is one of the most common tumors worldwide, with approximately 1.926 million new cases and 904,000 deaths globally in 2022 (1). In China, there were about 517,000 new cases and 240,000 deaths (2). Over 85% of CRC diagnoses occur at an advanced stage, and despite comprehensive treatments like surgery, radiation, chemotherapy, and targeted therapies, the five-year survival rate for CRC in China remains below 40%. However, if CRC is detected early, the five-year survival rate can be as high as 90% (3). Therefore, early detection of CRC and precancerous lesions is crucial for reducing incidence and mortality rates. In 2021, the incidence of colorectal cancer in Shanghai was 59.54 per 100,000, making it the most prevalent digestive system cancer. The significant burden of colorectal cancer poses serious threats to public health, highlighting the urgent need for effective prevention and control measures to curb its rising incidence.

Currently, most regions in China use a two-step screening strategy for community colorectal cancer screening, starting with a high-risk factor questionnaire and fecal occult blood testing (FOBT) as initial tests, followed by a full colonoscopy for further evaluation (4). Colonoscopy is the most accurate method for early detection of CRC, but it is an invasive procedure with associated risks of bleeding and perforation. Patients must also undergo bowel preparation, leading to poor adherence, with a colonoscopy compliance rate of around 20%, making it unsuitable for large-scale screening (5). Guaiac fecal occult blood testing (gFOBT) and fecal immunochemical testing (FIT) are two commonly used methods of FOBT, but they have low sensitivity and relatively high false positive rates (6).

In recent years, detecting stool DNA methylation from exfoliated colorectal cells has emerged as a potential screening method (7). Specific genes, including SDC2 (8), SFRP2 (9), TFPI2 (10), and ADHFE1 (11), have been found to be associated with colorectal CRC and precancerous lesions, while PPP2R5C can induce a G2/M blockade phenotype related to tumor development (12). The U.S. Food and Drug Administration(FDA) in 2014 approved the first stool-DNA colorectal screening test-The Cologuard test. It is a multi-target test that detects the presence of hemoglobin and colorectal cancer-related DNA mutations in shed abnormal cells using nine biomarkers. It can detect 92.3% of CRC cases and 42.4% of advanced adenomas, with a specificity of 86.7% (13). Currently, several domestic stool DNA methylation testing kits have demonstrated high sensitivity and specificity, but their clinical value for early screening of CRC and precancerous lesions in community populations requires further research.

To this end, we used stool DNA methylation detection (sDNAMD) for colorectal neoplasm (CN) screening in a real-world community setting, analyzing its impact on CN detection rates and colonoscopy compliance, and evaluating its application value in CN screening within a real-world community.

## 2 Materials and methods

### 2.1 Study population

According to the “Guidelines for Colorectal Cancer Screening and Early Diagnosis and Treatment in China” (14), between July 1, 2023, and June 30, 2024, residents aged 50 to 75 in the Maqiao Town community of Minhang District, Shanghai, were invited to voluntarily participate in colorectal cancer screening.

#### 2.1.1 Inclusion criteria

1) aged 50 to 75 years;2) No significant gastrointestinal symptoms; 3) Residents who had obtained informed consent; 4) Permanent residents of the Maqiao Town community of Minhang District, Shanghai.

#### 2.1.2 Exclusion criteria

1) Age <50 years or >75 years; 2)Adenoma or serrated polyp that has not been removed yet;3) Patients with colorectal cancer who have not undergone surgery or are highly suspected of having colorectal cancer through imaging and laboratory tests;4) Patients with or suspected of other malignant tumors;5) Hereditary CRC syndrome; 6) Coagulopathy and other severe comorbidities (dysfunction or failure of cardiopulmonary or renal function).

### 2.2 Random grouping

Individuals invited to participate in screening are assigned to one of the following three groups based on computer-generated numbers from their community. Until each group reaches the target number of people.

#### 2.2.1 High-risk questionnaire and FOBT

Participants completed a questionnaire assessing risk factors for CRC and underwent FOBT.

#### 2.2.2 High-risk questionnaire and FOBT and sDNAMD

A CRC high-risk questionnaire and FOBT testing were conducted for the enrolled participants. High-risk individuals and/or those with a positive FOBT result were further tested with sDNAMD.

#### 2.2.3 Stool DNA methylation detection

Participants underwent stool DNA methylation detection.

Based on the initial screening results, individuals in the three groups voluntarily underwent colonoscopy. The number of participants who underwent colonoscopy and the results were recorded, and the colonoscopy compliance rate and colorectal cancer detection rate were calculated.

### 2.3 Fecal sample collection and testing, colonoscopy, and tissue pathological diagnosis requirements

#### 2.3.1 FOBT sample and testing

Two fecal collection containers have been provided to the screened residents, with a one-week interval between the two collection times. The samples have been tested using the colloidal gold immunochromatographic method at the Maqiao Community Health Service Center within 4 hours of collection on the same day, and a test report has been provided. The minimum detectable amount of human hemoglobin in the fecal occult blood test kit(Hangzhou Biotest Biotech Co., Ltd) is 100ng/ml. The appearance of two purple red bands indicates that the concentration of human hemoglobin in the sample is above 100ng/ml, and the test result is positive; Only a purple red band appearing in the quality control area indicates a negative test result.

#### 2.3.2 sDNAMD sample and testing

Fecal samples of approximately 5g have been collected in a 50 ml tube, and 15 ml of preservative has been added according to the instructions of the fecal DNA methylation detection kit(Huachangkang, Huada Shuji Biotechnology (Shenzhen) Co., Ltd.). The samples have been stored at -80°C. Once fecal sample collection has been completed, the samples have been sent in batches to BGI Genomics for methylation level detection of the SDC2, ADHFE1, and PPP2R5C genes in the feces using quantitative PCR technology. A test result report has been provided.

Upon thawing, the stool samples were homogenized using a shaking device for 1 minute. They were then centrifuged at 12,000 x g per tube for 15 minutes. Genomic DNA was extracted using the TIA Namp Fecal DNA Kit, and sulfite conversion was performed with the DNA Sulfite Conversion Kit (Shenzhen BGI Gene Co., Ltd.) to screen for methylated DNA.

#### 2.3.3 Detect DNA concentration using a NanoDrop 2000 ultra-microvolume spectrophotometer (Thermo Fisher Technologies, USA)

After purification and transformation, store the DNA at -20°C for later use. Gene amplification is performed using a SLAN-96S real-time quantitative polymerase chain reaction (qPCR) instrument, and the experiment is repeated three times per sample. The methylation levels of three genes were quantitatively detected using the DNA methylation-specific polymerase chain reaction (MSP) assay kits for ADHFE1, SDC2, and PPP2R5C, with GAPDH serving as the internal reference gene.

#### 2.3.4 Colonoscopy

The colonoscopy has been performed by a physician with over five years of experience in colonoscopy at Shanghai Fifth People’s Hospital, Fudan University.

#### 2.3.5 Tissue pathology diagnosis

The gastrointestinal tissue pathology diagnoses have been carried out by a chief physician and an associate chief physician from the Pathology Department of Shanghai Fifth People’s Hospital, Fudan University.

### 2.4 Definition

#### 2.4.1 High-risk population

Individuals are classified as high-risk if they meet any one or more of the following criteria from the high-risk factor questionnaire: ① a first-degree relative with a history of colorectal cancer; ② a history of colorectal polyps; ③ a history of gallbladder or appendix surgery; ④ a history of chronic constipation or diarrhea, or rectal bleeding.

#### 2.4.2 FOBT positive

FOBT will be conducted twice, with a positive result determined if either one or both tests are positive; if both tests are negative, the result will be considered negative.

#### 2.4.3 Stool DNA methylation positive

The methylation status of 26 loci in the ADHFE1, SDC2, and PPP2R5C genes was detected. A result is considered positive if the number of methylated loci is ≥5, and negative if the number of methylated loci is <5.

#### 2.4.4 Colonoscopy positive

The colonoscopy result is considered positive for colorectal neoplasia, which includes colorectal cancer, adenoma, sessile serrated lesion, traditional serrated adenoma, and hyperplastic polyps ≥10 mm. A result with no colorectal neoplasia is considered negative. Dysplasia was defined as either low grade or high grade.

#### 2.4.5 Advanced adenoma

An AA was defined as an adenoma ≥10 mm or an adenoma with villous histology (≥25% villous) and/or high-grade dysplasia of any size.

#### 2.4.6 Serrated polyp

SP included sessile serrated lesion, traditional serrated adenoma, ≥5 mm proximal hyperplastic polyp and ≥10 mm hyperplastic polyp. Splenic flexure was the landmark to distinguish a proximal or distal location.

#### 2.4.7 Advanced serrated polyps

An ASP was defined as a serrated or hyperplastic polyp ≥1 cm and/or a serrated polyp with low- or high-grade dysplasia.

#### 2.4.8 Early colorectal cancer

This refers to cancer cells that are confined to the lamina propria or have penetrated the muscularis mucosa into the submucosa but have not invaded the muscularis propria.

### 2.5 Study outcomes

The primary outcome of this study is the detection rate of colorectal neoplasia in screening participants, calculated by dividing the number of detected cases of colorectal neoplasia by the number of individuals who underwent colonoscopy.

The secondary outcome is the colonoscopy adherence rate, calculated by dividing the number of individuals who received colonoscopy by the number of screening-positive participants. Positive predictive value (PPV %) = (Number of colorectal neoplasia cases detected by colonoscopy/Number of colonoscopy examinations) X 100%.

### 2.6 Statistical analysis

Statistical analysis was performed using SPSS (version 25.0). Chi-square tests and analysis of variance (ANOVA) were used to compare the differences in detection rates of colorectal neoplasia, colorectal polyps, advanced adenomas, colorectal cancer, and early-stage colorectal cancer across the three groups, as well as the differences in colonoscopy adherence rates. A p-value < 0.05 was considered statistically significant.

## 3 Results

### 3.1 Screening process and demographic distribution of age, gender, marital status, education, and occupation

**Figure 1:**
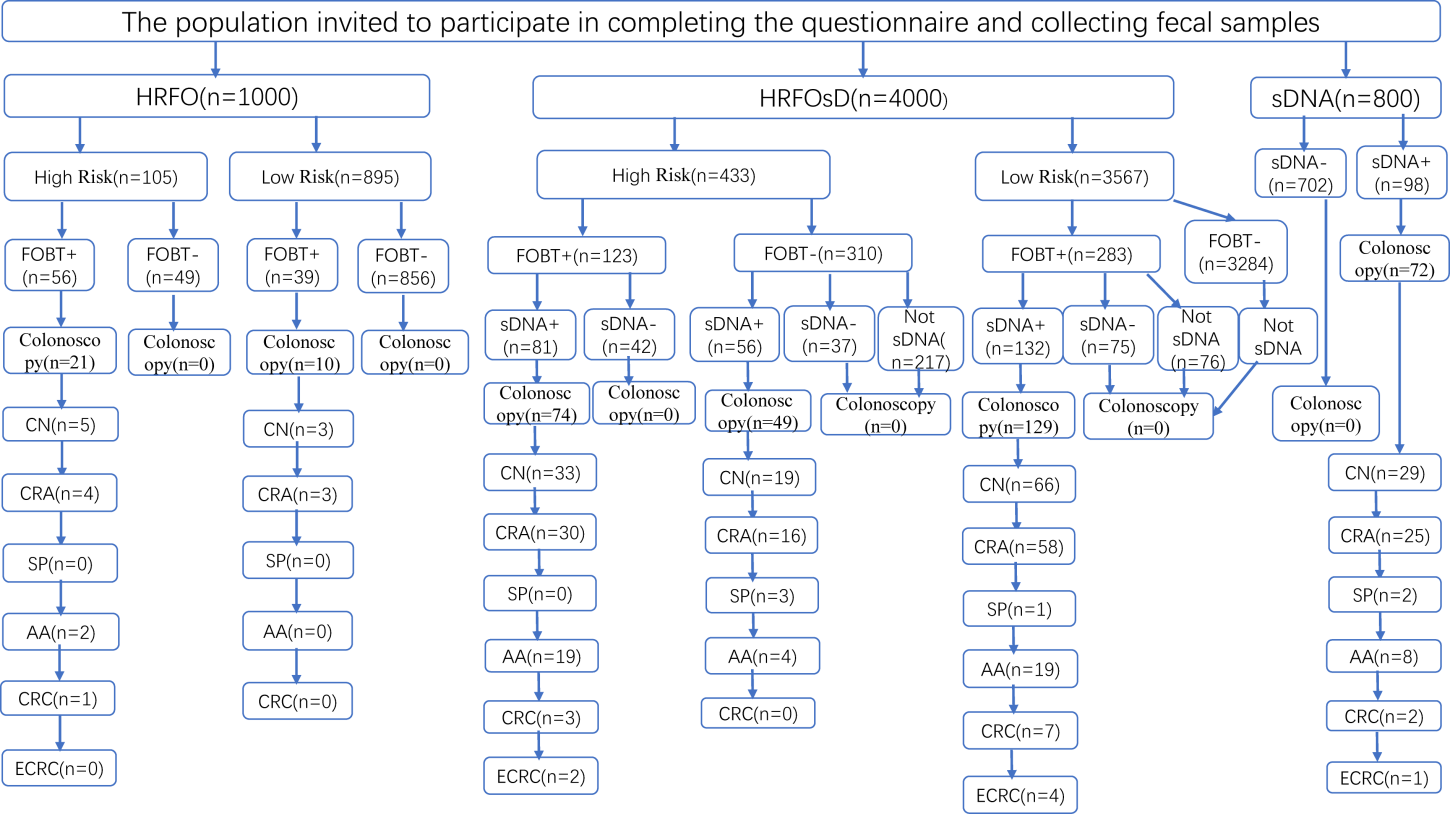
Flowchart of CRC screening and colonoscopy results. HRFO, High-risk questionnaire and fecal occult blood tests (FOBT); HRFOsD, High-risk questionnaire and FOBT and stool DNA methylation detection(sDNAMD). sDNA, stool DNA methylation detection; FOBT+, FOBT positive; FOBT-, FOBT negative. sDNA+, sDNAMD positive; sDNA-, sDNAMD negative; CN, colorectal neoplasia included CRC, adenoma, sessile serrated lesion, traditional serrated adenoma, and ≥10 mm hyperplastic polyp. CRA, Colorectal adenoma; SP, serrated polyp; AA, Advanced adenoma (An AA was defined as an adenoma ≥10 mm or an adenoma with villous histology (≥25% villous) and/or high-grade dysplasia of any size). CRC, Colorectal cancer; ECRC, Early Colorectal Cancer (This refers to cancer cells that are confined to the lamina propria or have penetrated the muscularis mucosa into the submucosa but have not invaded the muscularis propria).

The community CN screening process and colonoscopy results are shown in **Figure 1**. The HRFO group screened 1,000 individuals, with 463 men and 537 women, and an average age of 63.8 ± 6.8 years. In the HRFO group, both high-risk and low-risk residents, as well as those with a negative FOBT result, were unwilling to undergo colonoscopy. The HRFOsD group screened 4,000 individuals, with 1,799 men and 2,201 women, and an average age of 62.4 ± 6.7 years. The results showed that residents with negative sDNAMD results or those who did not undergo sDNA testing were unwilling to undergo colonoscopy. The sDNA group screened 800 individuals, with 362 men and 438 women, and an average age of 63.4 ± 6.5 years. All residents with negative sDNA results were unwilling to undergo colonoscopy. Marital status, education, and occupation distributions are shown in **Table 1**.

**Table 1:** Basic characteristics of individuals invited to complete colorectal cancer screening.

	HRFO	HRFOsD	sDNA
Cases n	1000	4000	800
Average age	63.8 ± 6.8	62.4 ± 6.7	63.4 ± 6.5
Year			
<65 n(%)	492(49.2)	2420(60.5)	435(54.4)
≥65 n(%)	508(50.8)	1580(39.5)	365(45.6)
Sex			
Male n(%)	463(46.3)	1799(45.0)	362(45.3)
Female n(%)	537(53.7)	2201(55.0)	438(54.7)
Marital status			
Married n(%)	980(98.0)	3907(97.6)	780(97.5)
Divorce n(%)	11(1.1)	44(1.1)	9(1.1)
Unmarried n(%)	3(0.3)	14(0.4)	1(0.1)
Widow n(%)	6(0.6)	35(0.9)	10(1.3)
Education			
High school and below n(%)	975(97.5)	3870(96.7)	771(96.4)
Junior college or below n(%)	11(1.1)	60(1.5)	21(2.6)
Bachelor degree or above n(%)	14(1.4)	70(1.8)	8(1.0)
Occupation			
Government organs and institutions n(%)	11(1.1)	46(1.2)	9(1.1)
Agriculture and commerce n(%)	490(49.0)	1210(30.3)	268(33.5)
Professional technology and clerks n(%)	6(0.6)	29(0.7)	9(1.1)
Unknow n(%)	493(49.3)	2715(67.8)	514(64.3)

HRFO, High-Risk Questionnaire and fecal occult blood tests (FOBT). HRFOsD, High-Risk Questionnaire and FOBT and stool DNA methylation detection (sDNAMD). sDNA, Stool DNA Methylation Detection.

### 3.2 Comparison of screening positivity rate and colonoscopy adherence

**Figure 2:**
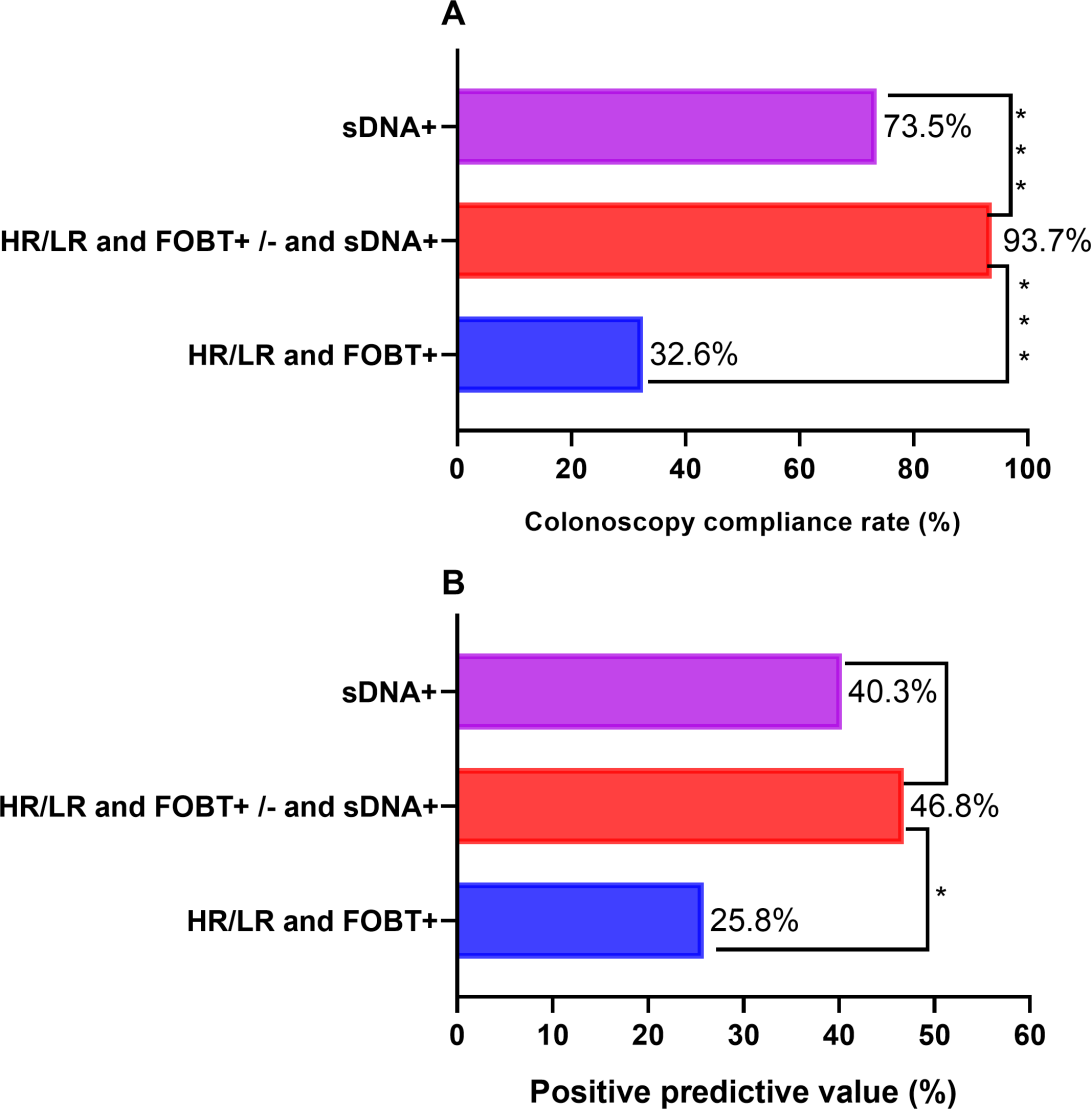
Comparison of compliance rate and colorectal neoplasia positive predictive value of colonoscopy. **(A)** The compliance rate of colonoscopy in the HR/LR and FOBT+/- and sDNA+groups was significantly higher than that in the HR/LR and FOBT+group and sDNA+group. **(B)** The colorectal neoplasia positive predictive value of colonoscopy in HR/LR and FOBT+/- and sDNA+groups was significantly higher than that in HR/LR and FOBT+groups. HR, High risk; LR, Low risk; FOBT+, fecal occult blood tests (FOBT) positive; FOBT-, FOBT Negative; sDNA+, stool DNA methylation detection positive.

**Table 2** shows that the proportion of high-risk individuals in the HRFO group was 10.5%, with a FOBT positivity rate of 9.5%. In the HRFOsD group, the proportion of high-risk individuals was 10.8%, with a FOBT positivity rate of 10.2%. No significant difference was found between the two groups. The sDNAMD positivity rate in the HRFOsD group was 63.6%, while in the sDNA group, it was 12.3%. A significant statistical difference was observed between the two groups (P<0.0001). The colonoscopy adherence rate was significantly different among the HRFOsD group (93.7%), HRFO group (32.6%), and sD group (73.5%) (**Table 2**, **Figure 2**; P<0.0001). The addition of fecal DNA methylation testing to the high-risk questionnaire and FOBT screening significantly improves colonoscopy adherence and CN positivity rate.

**Table 2:** Comparison of screening positive rate and colonoscopy results.

	HRFO	HRFOsD	sDNA	X²	*P*
HRQ					
HR n(%)	105(10.5)	433(10.8)	/	0.088	0.767
LR n(%)	895(89.5)	3567(89.2)			
FOBT					
FOBT+ n(%)	95(9.5)	406(10.2)	/	0.375	0.54
FOBT- n(%)	905(90.5)	3594(89.8)			
sDNAMD					
Positive n(%)	/	269(63.6)	98(12.3)	347.285	<0.0001
Negative n(%)		154(36.4)	702(87.7)		
Colonoscopy test					
Yes n(%)	31(32.6)	252(93.7)	72(73.5)	151.237^a^	<0.0001
No n(%)	64(67.4)	17(6.3)	26(26.5)	28.366^b^	<0.0001
Colonoscopy Results					
CN n(%)	8(25.8)	118(46.8)	29(40.3)	4.937^a^	0.026
No CN n(%)	23(74.2)	134(53.2)	43(59.7)	0.969^b^	0.325

HRQ, High-Risk Questionnaire; HR, High risk; LR, Low risk; FOBT+, fecal occult blood tests (FOBT) positive; FOBT-, FOBT Negative. a: Comparison between HRFOsD group and HRFO group. b: Comparison between HRFOsD group and sDNA group.

### 3.3 Subgroup comparison of colonoscopy adherence and CN detection rate

**Table 3** and **Figure 2** show that adding fecal DNA methylation testing to the high-risk questionnaire and FOBT screening significantly improves colonoscopy adherence and CN positive predictive value. Compared to the HR and FOBT+ subgroup, the LR and FOBT+ and sDNA+ subgroup showed a significantly higher detection rate of CN and colorectal adenomas. Regarding the CN detection rate, although there was no statistical difference between the HR and FOBT+ subgroup and the HR and FOBT+ and sDNA+ (*P*=0.086) and HR and FOBT- and sDNA+ (*P*=0.227) subgroups, the detection rates were 44.6% and 38.3%, respectively, which were notably higher than the 23.8% detection rate in the HR and FOBT+ subgroup.

**Table 3:** Comparison of compliance rate and detection rate of colonoscopy.

	HR and FOBT+	LR and FOBT+	HR and FOBT+ and sDNA+	HR and FOBT- and sDNA+	LR and FOBT+ and sDNA+	sDNA+
Cases N	56	39	81	56	132	98
Colonoscopy test						
n	21	10	74	49	129	72
CCR (%)	37.5	25.6	91.4	87.5	97.7	73.5
X²	Ref	1.471	45.177	29.867	88.436	19.275
*P*		0.225	<0.0001	<0.0001	<0.0001	<0.0001
Colonoscopy Results						
CN positivity rate n(%)	5(23.8)	3(30.0)	33(44.6)	19(38.3)	66(51.2)	29(40.3)
X²	Ref	0.136	2.945	1.461	5.42	1.901
*P*		0.713	0.086	0.227	0.02	0.168
CRA positivity rate n(%)	4(19.0)	3(30.0)	30(40.5)	16(32.7)	58(45.0)	25(34.7)
X²	Ref	0.465	3.288	1.333	5.001	1.861
*P*		0.495	0.07	0.248	0.025	0.172
AA positivity rate n(%)	2(9.5)	0	19(25.7)	4(8.2)	19(14.7)	8(11.1)
X²	Ref	/	2.478	0.035	0.406	0.043
*P*			0.115	0.852	0.524	0.836
*CRC positivity rate n(%)*	1(4.8)	0	3(4.1)	0	7(5.4)	2(2.8)
X²	Ref	/	0.02	/	0.016	0.205
*P*			0.887		0.9	0.651

HR, High risk; LR, Low risk; FOBT+, fecal occult blood tests (FOBT) positive; FOBT-, FOBT Negative; sDNA+, stool DNA methylation detection positive; CCR, Colonoscopy compliance rate; CN, Colorectal neoplasia; CRA, Colorectal adenoma; AA, Advanced adenoma; CRC, Colorectal cancer.

### 3.4 The impact of age and gender on colorectal neoplasia detection rate

**Table 4** shows that in both the HRFO and sDNA groups, age (grouped by 65 years) and gender had no significant impact on the colorectal neoplasia detection rate. In the HRFOsD group, however, the CN (*P*=0.022) and colorectal adenoma (*P*=0.044) detection rates were higher in females than in males, and age (grouped by 65 years) had no significant impact on the CN detection rate.

**Table 4:** The influence of gender and age on the detection rate of colorectal tumors.

	Colonoscopy(n)	CN	CRA	AA	CRC	ECRC
HRFO						
Sex						
Male n(%)	11	3(27.3)	2(18.2)	1(9.1)	1(9.1)	0
Female n(%)	20	5(25.0)	5(25.0)	1(5.0)	0	0
**X²**		0.019	0.189	0.197	1.879	/
***P***		0.890	0.664	0.657	0.170	
Year						
<65 n(%)	19	6(31.6)	5(26.3)	1(5.3)	1(5.3)	0
≥65 n(%)	12	2(16.7)	2(16.7)	1(8.3)	0	0
**X²**		0.854	0.392	0.115	0.653	/
***P***		0.355	0.531	0.735	0.419	
HRFOsD						
Sex						
Male n(%)	111	43(38.7)	38(34.2)	17(15.3)	4(3.6)	2(1.8)
Female n(%)	141	75(53.2)	66(46.8)	25(17.7)	6(4.3)	4(2.8)
**X²**		5.210	4.051	0.261	0.069	0.286
***P***		0.022	0.044	0.610	0.792	0.593
Year						
<65 n(%)	156	72(46.2)	64(41.0)	24(15.4)	5(3.2)	5(3.2)
≥65 n(%)	96	46(47.9)	40(41.7)	18(18.8)	5(5.2)	1(1.0)
**X²**		0.074	0.010	0.485	0.626	1.197
***P***		0.785	0.920	0.486	0.429	0.274
sDNA						
Sex						
Male n(%)	39	15(38.5)	14(35.9)	5(12.8)	0	0
Female n(%)	33	14(42.4)	11(33.3)	3(9.1)	2(6.0)	1(3.0)
**X²**		0.117	0.052	0.252	2.431	1.198
***P***		0.733	0.820	0.616	0.119	0.274
Year						
<65 n(%)	36	11(30.6)	9(25.0)	4(11.1)	1(2.8)	1(2.8)
≥65 n(%)	36	18(50.0)	16(44.4)	4(11.1)	1(2.8)	0
**X²**		2.289	3.003	0.000	0.000	1.014
***P***		0.093	0.083	1.000	1.000	0.314

HRFO, High-Risk Questionnaire and fecal occult blood tests (FOBT); HRFOsD, High-Risk Questionnaire and FOBT and stool DNA methylation detection (sDNAMD); sDNA, Stool DNA Methylation Detection; CN, Colorectal neoplasia; CRA, Colorectal adenoma; AA, Advanced adenoma; CRC, Colorectal cancer; ECRC, Early colorectal cancer.

## 4 Discussion

Early colorectal cancer and colorectal precancerous lesions are associated with three major genetic mechanisms: first, chromosomal instability caused by mutations in APC, KRAS, and TP53; second, microsatellite instability resulting from the functional loss of mismatch repair genes; and third, DNA methylation, an epigenetic change that leads to promoter hypermethylation and subsequent gene transcription suppression (15). The theoretical basis for stool DNA testing is that benign tumors and malignant lesions shed sufficient molecular material to be excreted in the feces, which can be detected through amplification techniques (16). Since sDNAMD is not restricted by specific diets or medications, it is non-invasive and tends to have higher patient compliance (17).

Recent studies have shown that the sensitivity of fecal SDC2 methylation for CRC screening ranges from 77.0% to 93.9%, with specificity between 88.2% and 98.1% (18, 19). The sensitivity of fecal SFRP2 detection for CRC screening is reported to be between 77% and 90%, with a specificity of 77%, demonstrating excellent sensitivity but unsatisfactory specificity (20). Additionally, Ahlquist et al. reported on the detection of methylation of several genes (vimentin, NDRG4, BMP3, and TFPI2) and KRAS mutations in fecal DNA, showing a sensitivity of 85% and specificity of 90% for CRC and colorectal adenomas (21). Among DNA methylation markers in CRC, abnormal methylation of SDC2 occurs in nearly all CRC tissues, regardless of stage, with elevated expression in various precancerous lesions and no detectable expression in normal intestinal mucosal tissue. The level of SDC2 methylation in tissue samples tends to increase with the severity of the lesions (22). Abnormal methylation of the ADHFE1 promoter is also observed in CRC, with the methylation status of the ADHFE1 promoter CpG island significantly higher in CRC tissues compared to normal mucosa (23).

Our study results show that adding sDNAMD screening to the high-risk questionnaire and FOBT screening can effectively improve actual community colonoscopy adherence. The colonoscopy adherence rate in the HRFOsD group (93.7%) was significantly higher compared to the HRFO group (32.6%) and the sDNA group (73.5%) (P<0.0001). For residents with negative FOBT results, negative sDNA results, or who did not undergo sDNA testing, there was no willingness to undergo colonoscopy. The reasons for this lack of willingness are likely related to the residents being asymptomatic, perceiving themselves as healthy, as well as fear and anxiety about the colonoscopy procedure, and difficulties with accepting the bowel preparation laxatives. In the HRFOsD group, the CN detection rate was higher in females than in males, which may be due to the fact that significantly more females participated in the HRFOsD group than males. Compared to the HR and FOBT+ group, the CN detection rate in the LR and FOBT+ and sDNA+ group was significantly higher. Adding sDNAMD to the high-risk questionnaire and FOBT screening significantly improves the CN detection rate.

As one of the most common non-invasive screening methods for CRC, FIT is more readily available and less expensive than multi-target stool DNA testing and colonoscopy screening. Compared to the non-invasive CRC screening tests available with other commercial products, FIT is more suitable for screening large populations (24). Compared with using HRFO alone, although HRFOsD and sDNA have increased detection costs, they can significantly improve colonoscopy compliance. HRFOsD can significantly improve CN detection rate, while sDNA alone does not significantly improve CN detection rate. The DNA positive CN detection rate is FOBT positive (44.6%) and negative (38.3%) in high-risk questionnaires, and FOBT positive (51.2%) in low-risk questionnaires. Therefore, we believe that conducting a questionnaire survey combined with FOBT testing in the community, followed by fecal DNA testing, and finally colonoscopy examination is a suitable solution for colorectal cancer screening in Chinese communities, which can balance screening costs and screening efficiency. We have found that long-term tracking and follow-up of community screening populations face many uncertain challenges, such as population mobility, compliance with follow-up, and the cost of long-term follow-up. With the development of artificial intelligence, establishing big data artificial intelligence for tracking and follow-up is the future direction.

Research has shown that in an 18-year follow-up, annual gFOBT screening reduced CRC incidence by 20%, while biennial gFOBT screening reduced CRC incidence by 17% (25). Studies have indicated that FIT screening lowers CRC mortality by 62% (26), and a meta-analysis shows that the sensitivity of FIT for detecting CRC is 79%, with a specificity of 94% (27). Stool DNA methylation detection has a sensitivity of 92.3% and a specificity of 86.6% for CRC and advanced adenomas (13). Currently, there is no data on the impact of Stool DNA methylation detection screening on reducing CRC incidence and mortality, and we will continue to track and observe our results.

The strength of our study lies in the fact that adding fecal DNA methylation testing to the high-risk questionnaire and FOBT screening significantly improves actual community colonoscopy adherence and the colorectal neoplasia detection rate. However, a limitation of the study is that residents with negative fecal DNA methylation results were unwilling to undergo colonoscopy, and there is a lack of analysis regarding the risk of missed colorectal neoplasia diagnosis in those with negative fecal DNA methylation results.

## Data Availability

The original contributions presented in the study are included in the article/supplementary material. Further inquiries can be directed to the corresponding authors.
